# Editorial: Addressing large scale computing challenges in neuroscience: current advances and future directions

**DOI:** 10.3389/fninf.2024.1534396

**Published:** 2024-12-17

**Authors:** Tam V. Nguyen, Min Wang, Domenico Maisto

**Affiliations:** ^1^Department of Computer Science, University of Dayton, Dayton, OH, United States; ^2^School of Information Technology and Systems, University of Canberra, Canberra, ACT, Australia; ^3^Institute of Cognitive Sciences and Technologies, National Research Council of Italy, Rome, Italy

**Keywords:** neuroscience, neural network, large-scale computing systems, high-performance computing (HPC), neuroinformatics infrastructure

## 1 Introduction

Neuroscience research generates vast amounts of data, requiring advanced computing resources for storage, management, analysis, and simulation (Glasser et al., 2016). Efficient utilization of high-performance computing architectures to process these massive datasets poses significant challenges, demanding the development of innovative computational methods and algorithms (Gorgolewski et al., 2011; Ding et al., 2018). Integrating advanced techniques is essential to address these issues, the integration of enabling researchers to overcome barriers and uncover new insights into brain function and structure (Markram et al., 2015; Eickenberg et al., 2017). This Research Topic highlights recent advancements and future directions in large-scale computing for neuroscience. The scope of this Research Topic included, among others

- Interdisciplinary strategies and collaborations to address issues related to data sharing, integration, and analysis.
- Security and privacy challenges, such as ethical issues, regulation, and government policies, and the potential for harmful or accidental data breaches.
- Reproducibility and transparency concerns in large-scale computing, including data sharing, standards, and best practices for data collection, analysis, and archival.
- Novel strategies and algorithms to exploit large-scale computing architectures and cloud technologies in neuroscience, e.g., for simulation, analysis, and data presentation.

This Research Topic provides a broad overview of the current challenges and emerging solutions, offering guidance for improving the scalability, efficiency, and accessibility of computational tools in this field. Four papers have ultimately been included; each one deals with one currently challenging aspect of large-scale computing in neuroscience.

## 2 The Papers

The four papers featured in this Research Topic explore these themes from different angles, presenting diverse strategies to advance data processing, simulation, and modeling in neuroscience. Two papers were Original Research papers, and two were Methods papers.

In the first Method paper, Villarreal-Haro et al. introduced CACTUS (Computational Axonal Configurator for Tailored and Ultradense Substrates), a computational workflow for generating white-matter substrates with predefined histological features of interest. The proposed three-step algorithmic procedure can generate synthetic axon populations with unprecedented biological fidelity. Achieving packing densities up to 95% of intracellular volume fractions and supporting voxel sizes up to 500 μm, CACTUS reproduces complex synthetic fibre configurations with biological plausibility that can be used as a numerical phantom to validate diffusion-weighted magnetic resonance images (DW-MRI) models. This enables more accurate modeling of diffusion-weighted magnetic resonance images. CACTUS represents a vital step toward bridging the gap between microscopic tissue properties and macroscopic imaging data.

Classifying neuron types from extracellular recordings is a cornerstone of neuroscience but remains constrained by traditional waveform-based methods. In the first Original Research paper, Haynes et al. introduce a machine learning-based approach to demix extracellularly recorded action potentials (EAPs), uncovering underlying spatial and temporal features that reflect neuronal morphology and electrophysiology. The authors developed a hierarchical classification system which, by applying a tensor components analysis to the features extracted through multiresolution wavelet analysis, furnishes a low-dimensional representation for recorded units that best characterizes waveform patterns shared across a diverse population of cortical neuron-type families. This method provides robust, interpretable features for neuron-type identification, and it highlights the potential of machine learning to tackle long-standing challenges in neuronal classification, making it a powerful tool for large-scale neuronal studies.

Simulating biologically realistic brain models at scale is essential for advancing our understanding of neural dynamics. Despite their utility the current platforms often fall short in flexibility, scalability, and ease of use. In the second Original Research paper, Miedema and Strydis introduce ExaFlexHH which addresses limitations above with an exascale-ready, flexible library for simulating Hodgkin-Huxley (HH) models on Field-Programmable Gate Array (FPGA) platforms. Leveraging the dataflow programming paradigm, ExaFlexHH achieves high scalability and energy efficiency for simulating large-scale brain models with HH-like neurons. Interestingly, ExaFlexHH is designed to consider user-friendliness and compliance with NeuroML, an XML brain model description prominent in computational neuroscience. The tests demonstrated near-linear performance gains across multiple FPGAs and exceptional resource efficiency in GFLOPS per watt concerning the classical High-Performance Computing approaches ExaFlexHH sets the stage for future computational neuroscience breakthroughs by enabling scalable, high-performance brain simulations.

Finally, in the second Method paper, Mönke et al. introduce SyNCoPy, short for Systems Neuroscience Computing in Python, a Python package for analyzing large-scale electrophysiological data. Its trial-parallel workflows and out-of-core computation techniques make it suitable for both small-scale and high-performance computing systems. SyNCoPy's seamless interoperability with other software and adherence to established conventions like FieldTrip—a widely diffused open-source Matlab toolbox for advanced analysis of electrophysiological data - enhance its accessibility for scholars. Consequently, this package represents an effective merge of the importance of user-friendly with powerful tools for large-scale data analysis, following a design paradigm that could reveal crucial in accelerating progress in systems neuroscience.

## 3 Conclusion

The papers featured in this Research Topic demonstrate how advanced computational methods and algorithms could overcome some critical challenges in large-scale neuroscience research. At broader level, this Research Topics could serve as a means for addressing the demands of large-scale neuroscience and anticipate future directions in this research field will continue to evolve, focusing on developing interoperable, scalable, and energy-efficient computational tools.
